# HIV integration and the establishment of latency in CCL19-treated resting CD4^+^ T cells require activation of NF-κB

**DOI:** 10.1186/s12977-016-0284-7

**Published:** 2016-07-26

**Authors:** Suha Saleh, Hao K. Lu, Vanessa Evans, David Harisson, Jingling Zhou, Anthony Jaworowski, Georgina Sallmann, Karey Y. Cheong, Talia M. Mota, Surekha Tennakoon, Thomas A. Angelovich, Jenny Anderson, Andrew Harman, Anthony Cunningham, Lachlan Gray, Melissa Churchill, Johnson Mak, Heidi Drummer, Dimitrios N. Vatakis, Sharon R. Lewin, Paul U. Cameron

**Affiliations:** 1 Doherty Institute for Infection and Immunity, The University of Melbourne, Melbourne, Australia; 2 Department of Infectious Diseases, Alfred Health, Monash University, Melbourne, Australia; 3 Centre for Biomedical Research, Burnet Institute, Melbourne, Australia; 4 Centre for Virus Research, Westmead Millennium Institute, University of Sydney, Sydney, Australia; 5 Department of Microbiology, Monash University, Melbourne, Australia; 6 Department of Infectious Diseases, School of Medicine, Deakin University, Melbourne, Australia; 7 Division of Hematology–Oncology, Department of Medicine, David Geffen School of Medicine at UCLA, Los Angeles, CA USA; 8 University California Los Angeles (UCLA) AIDS Institute, David Geffen School of Medicine at UCLA, Los Angeles, CA USA

**Keywords:** HIV latency, CD4^+^ T cells, Integration, NF-κB, Chemokine signalling

## Abstract

**Background:**

Eradication of HIV cannot be achieved with combination antiretroviral therapy (cART) because of the persistence of long-lived latently infected resting memory CD4^+^ T cells. We previously reported that HIV latency could be established in resting CD4^+^ T cells in the presence of the chemokine CCL19. To define how CCL19 facilitated the establishment of latent HIV infection, the role of chemokine receptor signalling was explored.

**Results:**

In resting CD4^+^ T cells, CCL19 induced phosphorylation of RAC-alpha serine/threonine-protein kinase (Akt), nuclear factor kappa B (NF-κB), extracellular-signal-regulated kinase (ERK) and p38. Inhibition of the phosphoinositol-3-kinase (PI3K) and Ras/Raf/Mitogen-activated protein kinase/ERK kinase (MEK)/ERK signalling pathways inhibited HIV integration, without significant reduction in HIV nuclear entry (measured by Alu-LTR and 2-LTR circle qPCR respectively). Inhibiting activation of MEK1/ERK1/2, c-Jun N-terminal kinase (JNK), activating protein-1 (AP-1) and NF-κB, but not p38, also inhibited HIV integration. We also show that HIV integrases interact with Pin1 in CCL19-treated CD4^+^ T cells and inhibition of JNK markedly reduced this interaction, suggesting that CCL19 treatment provided sufficient signals to protect HIV integrase from degradation via the proteasome pathway. Infection of CCL19-treated resting CD4^+^ T cells with mutant strains of HIV, lacking NF-κB binding sites in the HIV long terminal repeat (LTR) compared to infection with wild type virus, led to a significant reduction in integration by up to 40-fold (range 1–115.4, *p* = 0.03). This was in contrast to only a modest reduction of 5-fold (range 1.7–11, *p* > 0.05) in fully activated CD4^+^ T cells infected with the same mutants. Finally, we demonstrated significant differences in integration sites following HIV infection of unactivated, CCL19-treated, and fully activated CD4^+^ T cells.

**Conclusions:**

HIV integration in CCL19-treated resting CD4^+^ T cells depends on NF-κB signalling and increases the stability of HIV integrase, which allow subsequent integration and establishment of latency. These findings have implications for strategies needed to prevent the establishment, and potentially reverse, latent infection.

**Electronic supplementary material:**

The online version of this article (doi:10.1186/s12977-016-0284-7) contains supplementary material, which is available to authorized users.

## Background

The major barrier to HIV cure in patients receiving combination antiretroviral therapy (cART) is the persistence of long-lived, latently infected, resting memory CD4^+^ T cells [1]. Understanding how HIV latency is established and maintained is critical to the development of novel approaches to eradicate HIV. Direct infection of resting CD4^+^ T cells in vitro is inefficient [2, 3]. In contrast, HIV integration and latency is established in resting CD4^+^ T cells in vivo [4], in tonsil explants [5], or following co-culture with endothelial cells [6] or dendritic cells [7], or following culture with chemokines that bind to the chemokine receptors CCR7, CXCR3 and CCR6 expressed on resting CD4^+^ T cells [8, 9].

The chemokine CCR7 is expressed on naïve and central memory CD4^+^ T cells and culturing with the CCR7-ligands CCL19 or CCL21 facilitates entry and integration of HIV into these resting CD4^+^ T cells with minimal virus production. In this model, resting CD4^+^ T cells 5 day post infection represent a stable latently infected population that respond to latency-reversing agents (LRA) in a similar pattern to CD4^+^ T cells from HIV-infected individuals on cART [10]. We therefore believe this is an ideal model to study the early events required for establishing latency.

Binding of chemokines to their specific Gi-coupled receptors leads to activation of the RhoA/GTPase, phosphatidylinositol 3-kinase (PI3K) and phospholipase C (PLC) pathways [11]. Binding of HIV glycoprotein (gp)120 to the HIV co-receptor CXCR4 can also activate the RhoA pathway in resting CD4^+^ T cells, activating cofilin leading to depolymerization of the cortical actin cytoskeleton, thus facilitating nuclear entry [3, 12]. We demonstrated similar cellular changes with the exogenous chemokine CCL19 [9]. However, in contrast to HIV infection of resting CD4^+^ T cells, where there is minimal integration of HIV [2, 3, 9], in chemokine treated cells there was efficient nuclear localization and HIV integration [8, 9].

Activation of PI3K leads to changes in nuclear factor kappa B (NF-κB), the Mitogen-Activated Protein kinases (MAPK), extra-cellular signal-regulated kinases 1 and 2 (ERK1/2) [13], p38, c-Jun N-terminal kinase (JNK) [14] and serine/threonine protein kinase (Akt) phosphorylation (Additional file 1: Figure S1). Although NF-κB is a potent activator of HIV transcription, previous studies have demonstrated that latent or productive infection may depend on the relative amount of active NF-κB. In T cell lines, high levels of NF-κB enhance HIV transcription, but low levels of NF-κB are important for HIV integration [15]. Furthermore, generating latent infection by direct infection of Jurkat T cells requires low levels of NF-κB [16]. However, the role of NF-κB in latency in primary resting CD4^+^ T cells remains unknown.

Other downstream signalling products of chemokine receptor signalling may also impact HIV integration and latent infection of resting CD4^+^ T cells. Activating protein-1 transcription factors (AP-1) [17] may play a role in establishing and maintaining HIV latency [18]. The Ras/Raf/MEK pathway has previously been associated with the nuclear import of the HIV reverse transcriptase complex (RTC) [19] and thus may enhance HIV nuclear entry required for infection of resting CD4^+^ T cells. In the present study, we asked whether the PI3K signalling pathway, induced by ligation of CCL19 to CCR7, played a key role in the establishment of latent infection in CCL19-treated resting CD4^+^ T cells. We demonstrated that activation of the NF-κB pathway is critical for efficient integration of HIV in CCL19-treated resting CD4^+^ T cells and that the sites of HIV integration depended on the activation state of the cell at the time of infection.

## Results

### Dose response of CCL19 on resting CD4^+^ T cell signalling

We first determined the dose response of CCL19 on activation of downstream signalling in resting CD4^+^ T cells. After only 5 min of treatment with CCL19, we saw a strong increase in the level of phosphorylated Akt and NF-κB with all doses (range 30–300 nM) of CCL19 (Additional file 2: Figure S2A). However, we observed a dose response of CCL19 on the level of phosphorylated ERK. We believe that this may be due to different kinetic of activation. Indeed, treatment of resting CD4^+^ T cells with various doses of CCL19 for 15 min led to phosphorylation of Akt, NF-κB, ERK and low levels of JNK (Additional file 2: Figure S2B). Since very low levels of CCL19 (10–100 nM) are required for priming of resting CD4^+^ T cells for latent infection with HIV [8], we used 30 and 100 nM of CCL19 for subsequent infection and immunoblotting experiments, respectively.

### Inhibition of CCL19-induced signalling by pharmacological inhibitors

**Fig. 1:**
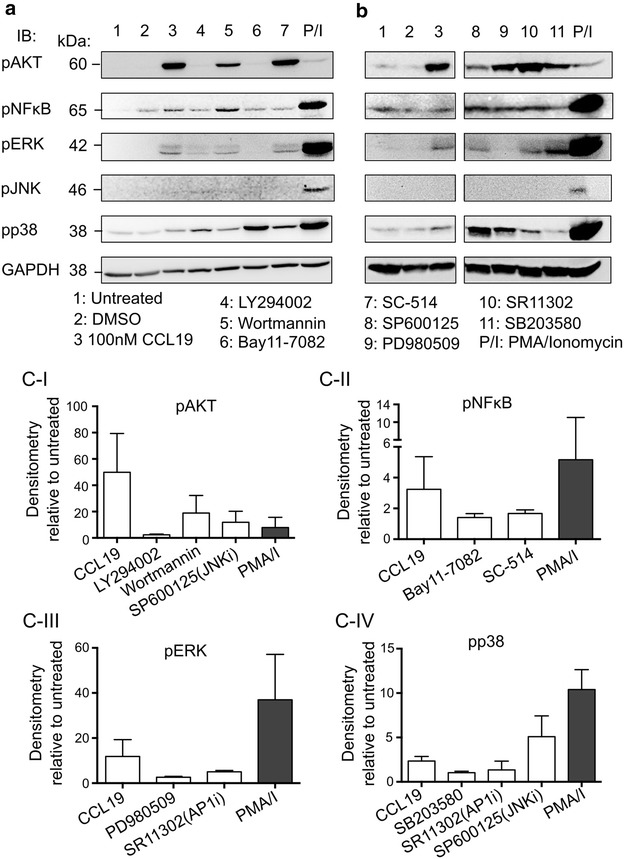
Inhibition of the CCL19-mediated signalling by pharmacological inhibitors. Resting CD4^+^ T cells were treated for 1 h with a predetermined (see “Methods”) concentration of inhibitor to PI3K (LY294002 and Wortmannin), NF-κB (Bay11-7082 and SC-514), JNK (SP600125), ERK (PD980509), AP-1 (SR11302) and p38 (SB203580) prior to the addition of CCL19 (100 nM) for 15 min. Cells were lysed and the level of specific phosphorylated proteins was measured using immunoblotting. **a**, **b** Representative immunoblots from two different donors treated with various inhibitors. GAPDH immunoblot was used as control for equal protein loading. **c** Densitometry of various phosphorylated proteins in the presence or absence of inhibitors in CCL19-treated resting CD4^+^ T cells. Values were normalised to both GAPDH and unactivated control. Data represent mean ± SD from 2 to 3 experiments

Activation of CCR7 has been shown to induce multiple modules of signalling leading to cell survival, chemotaxis, endocytosis and others (see review in [11, 20]). To determine which pathway of CCL19-mediated signalling was important for establishment of latency, pharmacological agents that inhibit various signalling molecules were used (Additional file 1: Figure S1). Inhibition of CCL19-induced phosphorylation of Akt, NF-κB, ERK and p38 were observed using specific inhibitors to PI3K (LY294002 and Wortmannin), NF-κB (SC-514) (Fig. 1a, c-I, c-II); MEK1/ERK1/2 (PD980509), p38 (SB203580) (Fig. 1b, c-III, c-IV); and a broad-spectrum inhibitor of Akt and NF-κB (Bay11-7082) (Fig. 1a, c-II). Phosphorylation of JNK was not consistently observed in response to CCL19. Since phosphorylation of JNK is required for cell migration [21], the lack of pJNK in CCL19-treated cells is likely due to timing of detection, as CD4^+^ T cell migration is normally measured at least 3 h after the addition of CCL19 [22]. However, the effect of JNK (SP600125) and AP-1 (SR11302) inhibitors on CCL19-treated cells were indirectly observed (Fig. 1c). SP600125 markedly inhibited phosphorylation of Akt, but increased p38 phosphorylation, while SR11302 inhibited the phosphorylation of both ERK and p38.

As expected, specific inhibition of PI3K (LY294002 and Wortmannin) and NF-κB activation (SC-514) had minimal effect on CCL19-mediated phosphorylation of ERK and p38 (Fig. 1a). Similarly, inhibition of the Ras/Raf/MEK signalling pathway had minimal effects on phosphorylation of Akt and NF-κB (Fig. 1b), demonstrating the dichotomy of these two pathways in CCR7-signalling.

### CCL19-mediated HIV integration is restricted by inhibitors of both PI3K and Ras/Raf/MEK signalling pathways

**Fig. 2:**
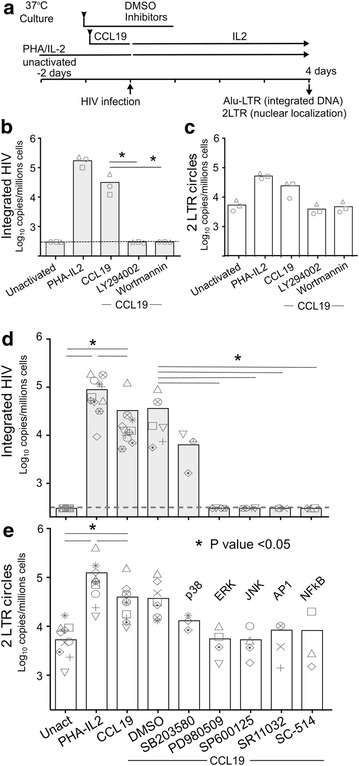
Treatment with PI3K and Ras/Raf/MEK inhibitors, eliminates HIV integration in CCL19-treated resting CD4^+^ T cells. **a** Resting CD4^+^ T-cells were pre-incubated with inhibitors of specific signalling pathways for 1 h before addition of CCL19, PHA-IL2 or DMSO and then infected with HIV NL4-3 for 2 h and cultured with media containing IL2 (1 U/mL) for up to 4 days following infection. HIV integration was measured by qPCR for Alu-LTR (**b**, **d**) and nuclear entry was measured by qPCR for 2-LTR circles (**c**, **e**). Experiments were also performed in the presence of inhibitors to PI3K (LY294002 and Wortmannin; **b**, **c**). **d**, **e** Further experiments were conducted in the presence of inhibitors of p38 (SB203580), ERK1/2 (PD980509), JNK (SP600125), AP-1 (SR11302), or NF-κB (SC-514) activation. Each *column* represents the mean copy number and the *symbols* represent individual donors. The detection limit for the Alu-LTR was 300 copies/10^6^ cells and is shown as a *dashed line*. *p < 0.05; **p < 0.005

We used inhibitors of the PI3K pathway, to determine if activation of this pathway was critical for HIV integration in CCL19-treated resting CD4^+^ T cells (Fig. 2). In HIV-infected CCL19-treated resting CD4^+^ T cells, treatment of cells prior to infection with the inhibitors of PI3K, LY294002 and Wortmannin, significantly reduced HIV integration as measured by Alu-LTR PCR, and reduced the levels of 2-LTR circles back to the level of unactivated cells (Fig. 2b, c).

To define the specific pathways downstream of the chemokine receptor important for HIV integration, we next tested inhibitors of p38, MEK1/ERK1/2, JNK, AP-1 and NF-κB, prior to infection of CCL19-treated resting CD4^+^ T cells. HIV integration was reduced to undetectable levels in the presence of inhibitors to MEK1/ERK1/2, JNK, AP-1 and NF-κB (Fig. 2d). In comparison, the p38 inhibitor SB203580 had a mild inhibitory (but not significant) effect on HIV integration (Fig. 2d), despite being able to fully inhibit p38 phosphorylation (Fig. 1). These inhibitors also reduced the level of 2-LTR circles back to unactivated cells level, although the reduction did not reach statistical significance (Fig. 2e). The effect of these inhibitors on HIV integration and 2-LTR circles were not likely due to the cytotoxicity of these drugs (Additional file 3: Figure S3). We found that the broad-spectrum inhibitor of Akt and NF-κB, Bay11-7082, was highly toxic to resting CD4^+^ T cells and was removed from further analysis.

### Pin1 is a cellular factor required for HIV integration in CCL19-treated cells

**Fig. 3:**
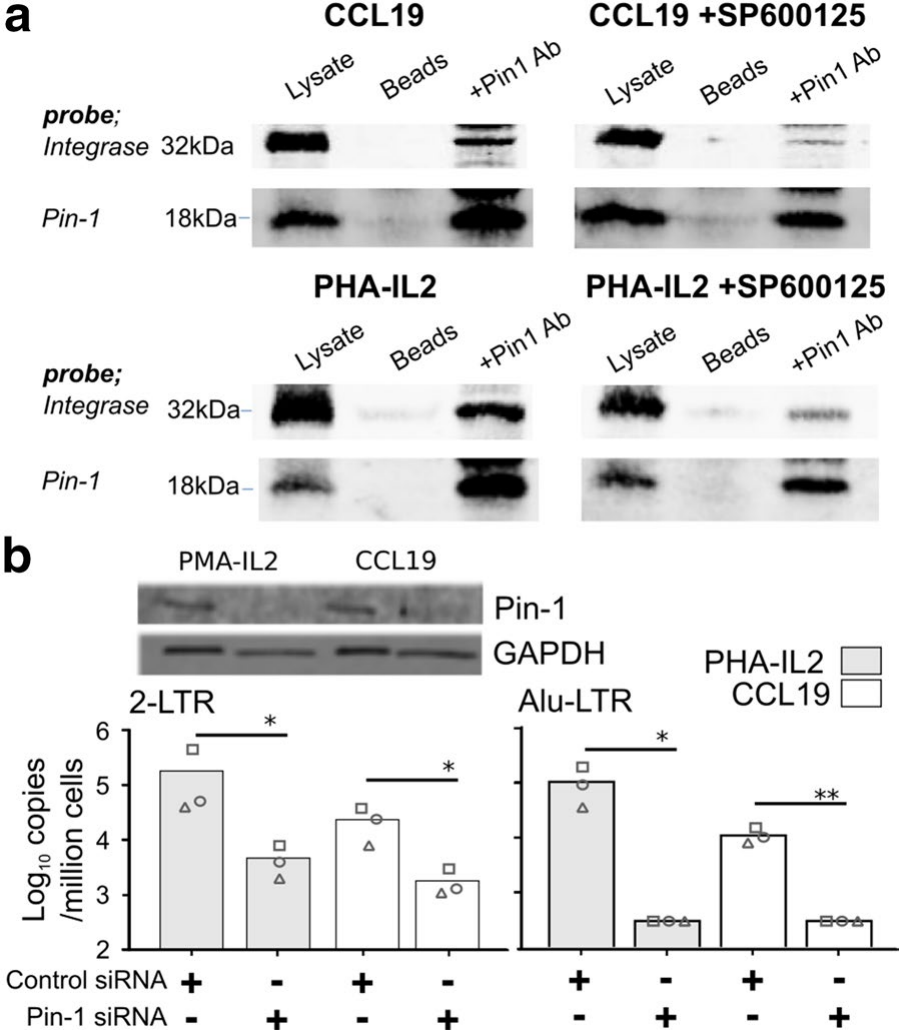
Role of Pin1 in HIV integration in CCL19-treated resting CD4^+^ T cells. Resting CD4^+^ T cells were cultured with CCL19 or activated with PHA-IL2 for 2 days prior to infection with HIV NL4-3. Immunoprecipitated proteins were analysed by SDS-PAGE and immunoblot with anti-HIV integrase antibody. **a** Co-IP of integrase and Pin1 was determined by IP with anti-Pin1 antibodies and probing for integrase (32 kDa) and Pin1 (18 kDa) in the presence and absence of the JNK inhibitor (SP600125, 10 µM) in HIV-infected CCL19-treated (*upper panel*) and PHA-IL2 activated (*lower panel*) CD4^+^ T cells. **b** PHA-IL2 activated and CCL19-treated resting CD4^+^ T cells were transfected with Pin1-specific or scrambled control siRNA, infected 2 days later with HIV, and 5 h later examined for Pin1 expression by immunoblot with antibodies to Pin1 or the GAPDH loading control (*lower panel*). The effects of Pin1 siRNA inhibition on HIV nuclear entry and integration were determined by real time PCR quantification of HIV 2-LTR circles (*left panels*) or Alu-LTR (*right panels*) respectively at day 4 post-infection. HIV-infected PHA-IL2 activated (*grey columns*) or CCL19-treated (*open columns*) CD4^+^ T cells are shown. Each *column* represents the mean of three donors. Individual donors are shown as *symbols*. *p < 0.05; **p < 0.01

Since JNK activation phosphorylates and stabilizes HIV integrase via an interaction with peptidyl prolyl-isomerase (Pin1) in activated CD4^+^ T cells [23], we used a co-immunoprecipitation (IP) assay to analyse the formation of Pin1/integrase-containing complexes in resting CCL19-treated CD4^+^ T cells. Like PHA-IL2 activation, CCL19 induced formation of Pin1/integrase complexes and this was impaired in the presence of the JNK inhibitor SP600125 (Fig. 3a). We also used siRNA knockdown of Pin1 to determine whether Pin1 was required for integration and establishment of latency in CCL19-treated resting CD4^+^ T cells. Expression of Pin1 in CD4^+^ T cells was similar after stimulation with PHA-IL2 or CCL19, but was greatly reduced in siRNA-transfected cells compared to the scrambled control (Fig. 3b, upper panel). Following HIV infection of these cells, we found a statistically significant reduction in both 2-LTR circles and integrated HIV DNA (Fig. 3b), consistent with a previous report [23]. These data suggest that Pin1 may affect pre-integration events in resting CD4^+^ T cells as well as PHA-IL2 activated cells. These experiments confirmed that interactions of JNK, integrase and Pin1 are likely similar following infection of resting and activated CD4^+^ T cells and are critical for HIV infection.

### NF-κB site 1 in the HIV LTR is essential for HIV integration in CCL19-treated resting CD4^+^ T cells

**Fig. 4:**
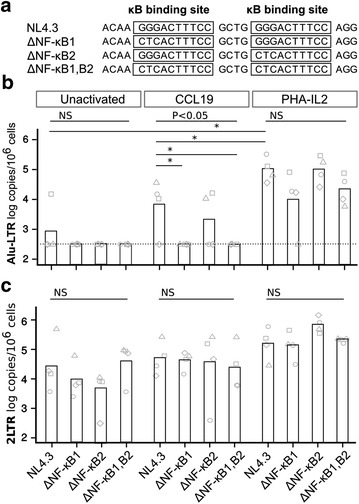
The NF-κB binding site in the HIV LTR is needed for efficient integration in CCL19-treated resting CD4^+^ T cells. **a** Mutations introduced into either or both NF-κB binding sites of the HIV LTR. The number of integrated HIV copies (**b**) and 2-LTR circles (**c**) per million cells 4 days following infection of unactivated, CCL19-treated and PHA-IL2 activated CD4^+^ T cells with wild type NL4-3 or NL4-3 containing mutations in the NF-κB binding sites, at both sites (ΔNF-κB1,B2) or either the first (ΔNF-κB1) or second (ΔNF-κB2) site alone. The *columns* represent the mean copy number and the individual donors are shown as different *symbols*. The detection limit for the Alu-LTR was 300 copies/10^6^ cells and is shown as a *dashed line*. The *upper line* and associated *p* value is for Kruskal–Wallis analysis of the four viruses and the *lower line* and *p* values shown as *asterisks* are for Mann–Whitney comparisons of two viruses or conditions. *p < 0.05, n = 4

To determine the role of NF-κB in the establishment of latency, we next infected CCL19-treated resting CD4^+^ T cells with HIV that contained a mutation in one or both NF-κB binding sites in the HIV LTR, which we refer to as ΔNF-κB1, ΔNF-κB2 or ΔNF-κB1,B2 respectively (Fig. 4a). No integration was detected after infection of CCL19-treated resting CD4^+^ T cells with NL4-3 ΔNF-κB1 or ΔNF-κB1,B2 (Fig. 4b), highlighting the critical role of the first NF-κB site in facilitating HIV integration in CCL19-treated resting CD4^+^ T cells. Infection with NL4-3 ΔNF-κB1,B2, compared to wild type NL4-3, showed a decreased in HIV integration to undetectable levels in all 4 donors by a mean (range) of 40 (1–115.4) fold (*p* = 0.03) in CCL19-treated CD4^+^ T cells with only a modest reduction in HIV integration of 5 (1.7–11) fold in fully activated CD4^+^ T cells although this was not significant. Similar results were obtained following infection with the ΔNF-κB1 single mutant when compared to wild type NL4-3. Infection with the ΔNF-κB2 single mutant showed a reduction in integration in CCL19-treated CD4^+^ T cells in only two of the 4 donors tested (Fig. 4b). In contrast, infection of PHA-IL2 activated CD4^+^ T cells resulted in a small but non statistically significant reduction in the levels of integration following infection with NL4-3 ΔNF-κB1 and ΔNF-κB1,B2 viruses (Fig. 4b).

In unactivated resting CD4^+^ T cells, integration was not detected (<300 copies/million cells, n = 4) for all viruses tested, except one donor where infection with NL4-3 wild type virus had detectable integration (Fig. 4b). There were no significant changes in the frequency of 2-LTR circles in the different culture conditions when the different mutant viruses were compared to wild type NL4-3 (Fig. 3c). Collectively, these data demonstrate that at least the first NF-κB binding site in the HIV LTR is required for efficient HIV integration in CCL19-treated resting CD4^+^ T cells.

### Identification of HIV integration sites in resting and activated CD4^+^ T cells

Given the importance of intact NF-κB sites for integration in CCL19-treated resting CD4^+^ T cells, we next asked if the site of HIV integration differed when HIV integration occurred in either resting or activated CD4^+^ T cells. Using inverse PCR and cloning, as previously described [24], we identified unique integration sites following HIV infection of CCL19-treated (number of unique clones, n = 247), PHA-IL2 activated (n = 432) and unactivated CD4^+^ T cells (n = 133). We compared the integration sites to those previously published in total CD4^+^ T cells from HIV-infected patients on suppressive cART [25], and from random sites in the genome. Using this method, we could not assess the frequency of the specific clonal integration sites as recently reported [26, 27], because we excluded analysis of integration sites that occurred more than once.

**Fig. 5:**
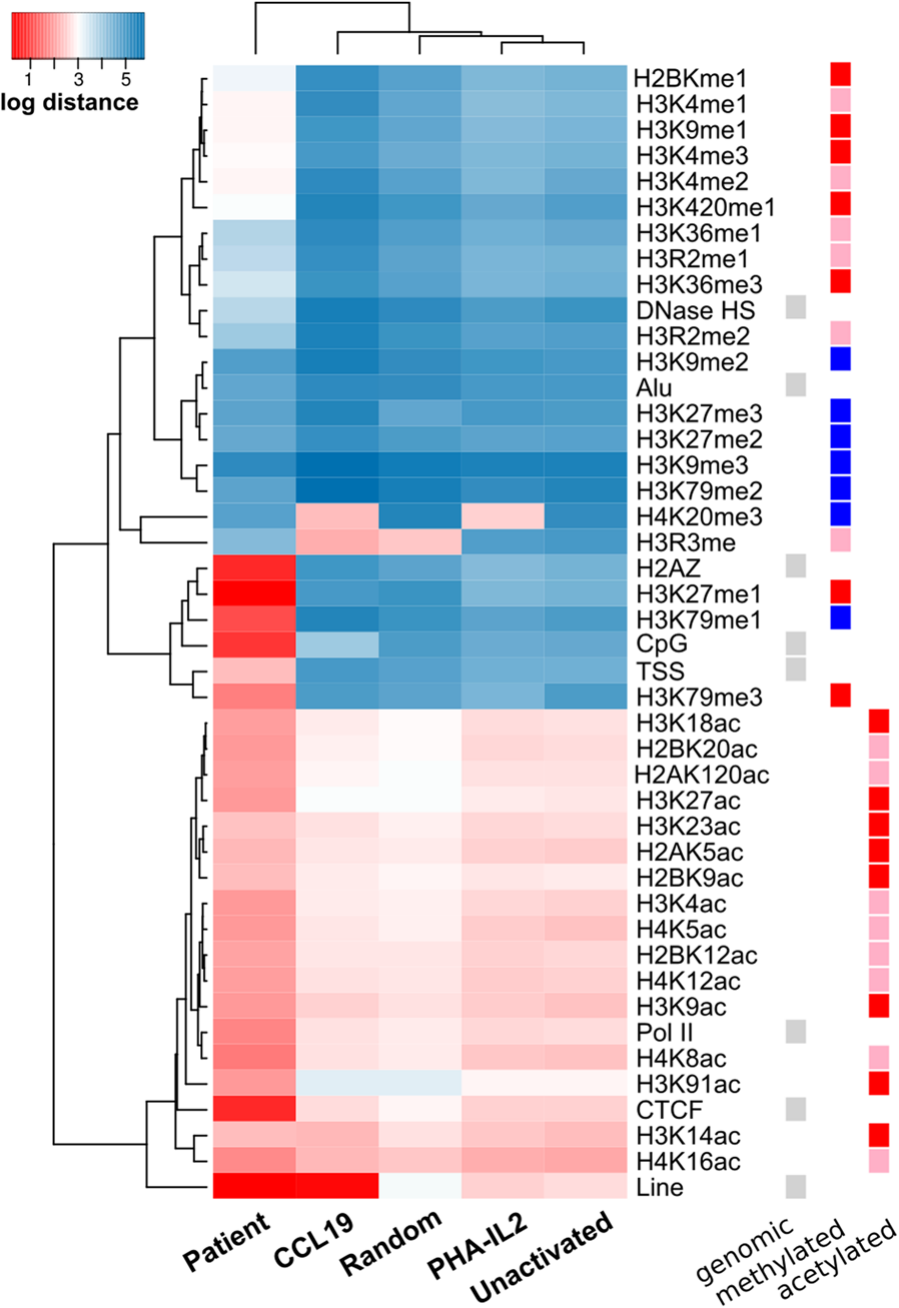
Distance of genomic features from the site of HIV integration. Following HIV infection of unactivated, CCL19-treated and PHA-IL2 activated CD4^+^ T cells, HIV integration sites were cloned, sequenced and mapped to the human genome. Genomic features were identified using the UCSC genome browser and the distance to the closest instance of each feature was determined. Comparison of the integration sites in each of the three in vitro conditions was made to integration sites previously described in CD4^+^ T cells from HIV-infected patients on cART (n = 4) [24] and randomly selected sites on the genome (n = 10,000; random). The distance for each site was analysed and plotted as log mean distance. *Blue* represents increased and red represents decreased distance from the genomic feature. The *labels* on the *y*-*axis* are shown in Additional file 1: Table S1. The *small boxes on the right of the figure* describe the main features of the integration sites and are classified as genomic (*grey*), methylated (associated with elevated transcription, *red*; intermediate transcription, *pink*; and repression, *blue*) and acetylated (associated with promoters, *pink*; associated with TSS, *red*) features

The distance from a series of genomic and epigenomic features are summarised as a heat map (Fig. 5). First, in all infection conditions, HIV preferentially integrated in actively transcribed genes (Additional file 4: Figure S4A, B) as previously reported for activated and resting CD4^+^ T cells [28, 29] and the Jurkat cell line [16]. Second, the distance from the genomic and epigenomic features in all in vitro models was similar, and these sites clustered separately from the patient-derived cells and random sites (Fig. 5). Finally, we found multiple differences between the integration sites in the different in vitro conditions, after adjusting for multiple comparisons (Additional file 5: Table S1).

Significant differences in integration sites between the different in vitro conditions were seen in the distance from the H2AZ, DNAse hypersensitivity site, Alu including Alu S, Alu J and Alu Y (Additional file 6: Figure S5A) and transcriptional start sites (TSS), which are all highly transcriptionally active regions. We saw no significant differences in the distance from CCCTC-binding Factor (T cell Factor, TCF), polymerase (pol) II, CpG and the majority of histone methylation and acetylation sites (Additional file 5: Table S1). For other genes such as Long Interspersed Nuclear elements (LINE 1), the integration sites in CCL19-treated infected cells were significantly closer than other in vitro conditions (Additional file 4: Figure S4C). This site tends to indicate gene rich regions. Based on the median distance from LINE 1, in CCL19-treated cells HIV showed a very strong preference to integrate in these sites, similar to patient derived cells. There was no association with culture conditions and proximity to RTE or CR1 but in CCL19-treated cells, HIV integrated closer to L1 sites and more distant from L2 (Additional file 6: Figure S5B). For histone methylation sites associated with intermediate transcriptional activity, in CCL19-treated cells HIV integrated further away than in PHA-IL2 and unactivated cells. The exception in this group was H4R3me2, a methylation site initially thought to be involved in priming gene expression but more likely mediating global gene repression in T-cells [30] (Additional file 5: Table S1). Other histone methylation sites were distant from integration sites, except for H4K20me3, which is strongly associated with heterochromatin [31]. Integration of HIV in CCL19-treated cells, as well as in PHA-IL2 fully activated cells, had a strong preference for this region (Additional file 4: Figure S4C, Additional file 5: Table S1).

We used gene array analyses to look at the gene expression in different in vitro conditions and sought to determine if there was a relationship between expression of a specific gene and the site of integration. There was no significant difference between the three conditions in the level of expression of genes at the HIV insertion sites (Additional file 4: Figure S4A). The majority of the genes where HIV integration occurred, were involved in cellular housekeeping activities (such as ubiquitin activity, nuclear transport, and nucleotide metabolism) and cell signalling pathways, and were expressed at similar levels in CCL19-treated and unactivated resting CD4^+^ T cells (Additional file 4: Figure S4B).

Finally, we have identified 152, 85 and 62 genes hosting integrated HIV in PHA-IL2, CCL19 and unactivated cells, respectively, of which 40 genes with insertion sites in two or more of the three cell populations (Additional file 7: Figure S6). Only two genes (TBCD; tubulin folding cofactor D and NSD1; nuclear receptor binding SET domain protein 1; Additional file 7: Figure S6, blue font) had been previously identified in infected CD4^+^ T cells from HIV-infected patients on cART [25–27, 32, 33]. We did find HIV integration in genes associated with mitosis and cancer, but these specific genes were not enriched in the three culture conditions (Additional file 7: Figure S6; red font). These data suggest that the selection for integration sites in CD4^+^ T cells from HIV infected patients on cART differs from in vitro infection of resting or activated CD4^+^ T cells.

## Discussion

We have previously shown that multiple chemokines facilitate efficient HIV nuclear localization and integration in resting CD4^+^ T cells in vitro [9]. Chemokines or HIV itself, via binding of gp120 to the co-receptor CXCR4, can activate the RhoA/Rac/cdc42 pathways and disrupt cortical actin to allow passage of the pre-integration complex into the nucleus [3]. However, in purely resting CD4^+^ T cells, integration remains inefficient even if nuclear localization occurs [3, 9]. We now demonstrate that CCL19 signalling, via the PI3K pathway and NF-κB, plays a key role in HIV integration in resting CD4^+^ T cells. HIV integration was reduced by inhibitors of NF-κB or upstream signalling pathways that reduce NF-κB activation. This is consistent with data from T cell lines, where low levels of NF-κB favored latent infection, while high levels of NF-κB favored productive infection [15].

We show that deletion of both NF-κB binding sites in the LTR eliminated HIV integration in CCL19-treated CD4^+^ T cells, but not in activated CD4^+^ T cells. Viruses with deletions or base-pair substitutions in the NF-κB enhancer of the LTR vary in replication competence in different in vitro models [34, 35]. Other studies have shown a direct correlation between viral replication and the level of NF-κB protein expressed in the specific cell that was infected [35]. Mutation of GGG to CTC (or TCT) in the NF-κB sites in the LTR abrogated NF-κB binding [36] and resulted in lower HIV replication than wild type virus [37]. Although we found a small reduction in the replicative capacity of the NF-κB mutated viruses in PHA/IL2 activated CD4^+^ T cells, as measured by integrated HIV DNA, there was no integration of mutant virus in CCL19-treated CD4^+^ T cells. Our data in a resting CD4^+^ T cell model of HIV latency shows a critical role for NF-κB in HIV integration in the CCL19-treated primary resting CD4^+^ T cells.

NF-κB, or indeed other transcription factors, could potentially increase efficient HIV integration in CCL19-treated resting CD4^+^ T cells in several ways. First, in other retroviruses, direct interaction of transcription factors with LTR enhancer sequences play a pivotal role in directing integration toward genomic regions rich in transcription factor binding sites [38]. In murine leukemia virus, transcription factors binding to the viral LTR enhancer may tether retroviral integrase and the pre-integration complex to transcriptionally active regulatory regions [39]. Second, NF-κB may facilitate HIV integration through its activity in homologous recombination and DNA repair, which is required for HIV integration [40]. The Lens epithelium–derived growth factor p75 splice variant (LEDGF) is a chromatin-binding protein that can direct HIV into active transcription units, and promotes the repair of DNA double-strand breaks by the homologous recombination repair pathway [41]. NF-κB may promote homologous recombination and DNA repair through interactions with Rad51, a key regulator of the homologous recombination pathway of DNA repair [42], and with the Breast cancer 1-C-terminal binding protein 1 (BRCA1-CtIP) complex, which accelerated Rad51 foci formation [42]. These observations, and our findings, suggest an important role for NF-κB activation in HIV integration, at least in resting CD4^+^ T cells that lack alternative transcription factors.

Other transcription factor binding sites, in particular AP-1, have been shown to be critical for the establishment of latency amongst different subtypes of HIV [18]. Here we show that CCL19 could induce the signalling cascade upstream of AP-1, indicating that chemokine signalling may also maintain latent infection after integration has occurred. However, the importance of AP-1 in maintaining HIV latency was not specifically tested in this model.

We also showed that akin to fully activated CD4^+^ T cells, CCL19 also induced signalling of the Ras/Raf/MEK/JNK pathway. Activated JNK was shown to bind and phosphorylate HIV integrase, which then interact with Pin1 [23]. Pin1 was shown to play a crucial role in stabilizing integrase, in the form of a pre-integration complex, and prevent its degradation via the proteasome pathway [23]. Thus inhibitors of the Ras/Raf/MEK/JNK signalling pathway could lead to significant impairment in HIV integration and 2-LTR circles formation in both CCL19 and PHA-IL2 treated CD4^+^ T cells. This is further supported by evidence that knockdown of Pin1 led to a similar effect as using inhibitors of the Ras/Raf/MEK/JNK signalling pathway on both HIV integration and 2-LTR circles formation. The reduction of 2-LTR circles to levels found in unactivated cells in the presence of PI3K or NF-κB inhibitors was less clear. One possible explanation was that phosphorylated NF-κB binds to its binding sites in the LTR and facilitates nuclear entry of the pre-integration complex (Fig. 4 and [43]), thus inhibition of NF-κB signalling could lead to a reduction of nuclear entry and subsequently affect both the level of integration and 2-LTR circles.

Finally, we show that the site of HIV integration differs between cells with differing activation states at the time of HIV infection. The majority of integration sites were within active transcriptional units, as previously shown for patient derived cells and other models of in vitro infection [24, 44, 45]. However, CCL19-treated resting CD4^+^ T cells had a pattern of integration that differed significantly from the other in vitro conditions, suggesting HIV integration site selection may depend on the balance of transcription factors, including NF-κB, at the time of infection. Differences in integration site may also contribute to the varied response to latency activators using different in vitro models of latent infection [10]. Our findings are in contrast to a recent study of multiple in vitro models of latency, where integration sites were associated with differences in virus expression in individual models, but integration sites and associated genomic features were not predictive across different models of latency [45]. It is important to note that the analysis used for our study was different because we compared infected resting and activated cells, rather than comparing expressed and inducible infection in different models of latency. In our method of integration site analysis we considered only unique sites in each condition and were therefore unable to show over-representation of the particular integration sites recently reported for BACH and other genes using CD4^+^ T cells from HIV-infected patients on cART [25–27, 32, 33].

## Conclusions

We show that activation of NF-κB and the NF-κB binding site of the HIV LTR enhancer promoter are critical for efficient HIV integration in CCL19-treated resting CD4^+^ T cells. CCL19 signalling increases the stability of HIV integrase, protects it from degradation, which allows subsequent integration and establishment of latency. Furthermore, we show significant differences in integration sites following HIV infection of unactivated, CCL19-treated, and fully activated CD4^+^ T cells. These findings have implications for strategies needed to prevent the establishment, and potentially reverse, latent infection.

## Methods

### Isolation of CD4 T cells

Peripheral blood mononuclear cells (PBMC) were isolated from buffy coats obtained from the Australian Red Cross Blood Service (Melbourne, Australia). Resting CD4^+^ T cells were isolated by magnetic bead depletion and cell sorting as previously described [8]. The resulting T cells consisted predominantly of naïve and central memory T cells [46] and purity was routinely >95 %, as assessed by flow cytometry.

### Cell lysis, immunoblotting (IB) and immunoprecipitation (IP)

To measure the effect of CCL19 on activation of the PI3K signalling pathway, we first determined the dose and kinetics of CCL19 on key signalling proteins phosphorylation in resting CD4^+^ T cells. In brief, freshly isolated resting CD4^+^ T cells were cultured overnight in RF1 [RPMI1640 media supplemented with 1× penicillin–streptomycin–glutamine (Life Technologies, Carlsbad, CA) and 1 % heat-inactivated fetal bovine serum (Bovogen Biologicals, Melbourne, Australia)]. On the following day, five million viable cells per condition was stimulated with various doses of CCL19 (30, 100, 200 and 300 nM) for exactly 5 or 15 min and immediately lysed with RIPA buffer (1 % NP40, 0.5 % SDS, 1 mM DTT, 10 mM Tris–HCl pH 7.6, 150 mM NaCl, 2 mM EDTA) containing Halt protease and phosphatase inhibitor cocktail (Thermo Fisher Scientific, Waltham, MA). Unstimulated or phorbol 12-myristate 13-acetate (PMA, 10 µg/ml Sigma-Aldrich, St. Louis MO) and ionomycin (2 µM, Sigma-Aldrich) stimulated cells were used as controls.

Protein concentrations of cell lysates were measured by the Bradford protein assay (Bio-Rad, Hercules, CA). Total cell lysates (50 μg) were resolved on a 10 % SDS-polyacrylamide gel, transferred onto nitrocellulose membrane, and processed further for immunobloting as previously described [9]. The membrane was sequentially probed with 1:1000 of anti-p-NF-κB (ser529, BD Biosciences, San Jose, CA), anti-p-Akt, anti-p-JNK, anti-p-ERK1/2, anti-p-p38, and anti-glyceraldehyde 3-phosphate dehydrogenase (GAPDH; all from Cell signalling Technologies, Danvers, MA), and re-probed with either horseradish peroxidase-conjugated anti-mouse or anti-rabbit secondary antibodies (Cell Signalling Technologies). The signal density was analysed using electrochemiluminescence (ECL) plus Western blotting detection reagents (GE Healthcare, UK) and MF-ChemiBIS chemiluminescence imaging (DNR Bio-Imaging systems, Jerusalem Israel).

Immunoprecipitation experiments were performed using 200 µg of total cell lysate from CCL19 or PHA-IL2_treated CD4^+^ T cells in the presence or absence of JNK inhibitor, SP600125 (10 µM, Sigma-Aldrich). Primary antibodies to Pin1 (Santa Cruz Biotechnology Inc, Dallas, TX) was added and incubated overnight at 4 °C followed by the addition of protein-G-sephrose beads (Genscript, Piscataway, NJ) for another 3 h. After washing the beads, the samples were subjected to IB analysis using anti-HIV integrase antibody (Abcam, Cambridge, UK).

### Inhibition of PI3K signalling pathway using pharmacological inhibitors

Freshly isolated purified CD4^+^ T cells were rested overnight in RF1 and incubated in the presence or absence of either PI3K inhibitors LY294002 (50 µM) and Wortmannin (100 nM); p38 inhibitor SB203580 (5 µM); MEK1/ERK1/2 inhibitor PD980509 (50 µM); JNK inhibitor SP600125 (10 µM, All from Sigma-Aldrich); NF-κB inhibitors SC-514 (100 µM) and Bay 11-7082 (10 µM, Calbiochem, San Diego, CA); or AP-1 inhibitor SR11032 (1 µM, Tocris Biosciences, Bristol, UK) for 1 h. Cells (5 million) were treated with 100 nM of CCL19 for 15 min, lysed and immunoblotted as described above. Unstimulated or phorbol 12-myristate 13-acetate (PMA, 10 µg/ml Sigma-Aldrich) and ionomycin (2 µM, Sigma-Aldrich) stimulated cells were used as controls. Protein intensity was analysed using densitometry of gel images using FIJI software [47].

### Plasmids and virus production

293T cells (ATCC, Manassas, VA) were transfected according to the manufacturer’s instructions (FuGene; Roche Diagnostics, Indianapolis, IN) with the plasmid coding for CXCR4 (X4)-using virus NL4-3 (kindly supplied by Prof. Damian Purcell, University of Melbourne, Australia). Viral plasmids with mutations in the NF-κB binding sites in the HIV long terminal repeat (LTR) were kindly provided by Dr. Monsef Benkirane (Institute of Human Genetics, Montpellier, France [48]) and included those with either a single mutation (GGG → CTC) at position −105 (site 1) or position −92 (site 2), or at both sites (double mutant) [49]. Following transfection, culture supernatants were concentrated over sucrose gradients and assessed for reverse transcriptase (RT) activity [8].

### Infection of resting CD4^+^ T cells

Resting CD4^+^ T cells were isolated by magnetic bead depletion and cell sorting, as previously described [8]. Purified cells were incubated with CCL19 (30 nM) for 24 h prior to infection. When pharmacological inhibitors of PI3K signalling were used, inhibitors were added 1 h prior to the addition of CCL19. Cells activated for 2 days with phytohemagglutinin (PHA, 10 μg/ml; Sigma) and IL2 (10 IU/ml, Roche Diagnostics) or left unstimulated were used as controls. Cells were then infected with NL4-3 virus, or with NL4-3 that had a mutation in one or both NF-κB binding sites at 1 reverse transcriptase (RT) count per minute (CPM)/cell for 2 h at 37 °C and washed before further culture with IL2 (1 IU/ml).

### Cytotoxicity assay

To address the toxicity of the pharmacological agents used, freshly isolated resting CD4^+^ T cells were treated with the same dose of pharmacological inhibitors in the presence of CCL19 (30 nM) for 48 h. Cells were then washed and cultured for a further 72 h. The percentage of viable cells on day 2 and day 5 was determined by flow cytometry using live fixable viability dye eFluor^®^ 780 (eBioscience, San Diego, CA), according to manufacturer’s instruction.

### Quantification of HIV infection

Following infection, RT was quantified in cell culture supernatant as previously described [8]. Integrated HIV DNA and 2-LTR circles were quantified using real time PCR (iCycler, Bio-Rad, Hercules, CA) as previously described [9, 50].

### Small interfering (si)RNA nucleofection

Resting CD4^+^ T cells were transfected with peptidyl prolyl-isomerase (Pin1) specific siRNA (sc-36230-SH; Santa Cruz, Biotechnology Inc, Dallas, TX) or control (scrambled) siRNA (sc-37007, Santa Cruz Biotechnology Inc), at 100 pmol per reaction using nucleofection (Amaxa Human T cell Nucleofector kit, Lonza, Cologne, Germany) according to the manufacturer’s instruction. Pin1 siRNA sequences: 5′-GUCAGAUGCAGAAGCCAUU-3′, 5′-CCGAAUUGUUUCUAGUUAG-3′, and 5′-UCCUCUGUUCAGUCGCAAA-3′. Knockdown of Pin1 protein was confirmed by Immunoblot using anti-Pin1 antibody (Santa Cruz Biotechnology Inc). Transfected cells were maintained in RPMI 1640 with 10 % Fetal calf serum for up to 48 h and stimulated with either PHA-IL2 or CCL19 prior to HIV infection or analysed by IB.

### Cloning and sequencing of HIV integration sites

Resting CD4^+^ T cells were activated with PHA-IL2, CCL19, or left unactivated before infection with HIV. Cells were harvested at day 4 post infection and the levels of viral integration were assessed by real-time PCR assay for Alu-LTR [50]. The identification of HIV integration sites was determined by an inverse PCR assay as previously described [24]. The integration site sequences were mapped onto the human genome using University of California Santa Cruz (UCSC) Genome Bioinformatics database. All the genomic feature data sets were downloaded from the UCSC genome database. The pattern of these features was compared between the in vitro conditions and CD4^+^ T cells from HIV-infected patients on antiretroviral therapy from published data [25]. To assess patterns in relation to genomic features, such as DNA methylation and/or acetylation pattern, the distance from these genomic features was calculated using Genomic Hyperbrowser (http://hyperbrowser.uio.no/hb/). Gene expression in the in vitro infected cells was determined using gene array analysis [51]. Cells were activated with CCL19 for either 6 h or 72 h before infection, gene expression from the two time-points were compared to unactivated or PHA-IL2 activated cells.

### Statistical analysis

Graphpad Prism was used for statistical analyses and preparing plots. A Kruskal–Wallis test was used for comparisons between groups and a Mann–Whitney test for analysis within groups. For comparisons between groups either a Student’s *t* test or a Mann–Whitney *U* test was used. Normalization was performed by log transformation before analysis.

The statistical program R [51] was used for analysis of gene arrays, cluster analysis and heatmap generation. A Student’s *t* test or Mann–Whitney test was used for comparisons between populations and *p* < 0.05 was considered significant.

For the site of integration, a Fisher’s exact test was used to determine the statistical significance between the groups when examining the proportion of integration sites that were near or far from a specific genomic feature. In addition, we treated the median distance of integration sites as a measure of association for that genomic feature. Since the populations of integration sites failed the normality tests, we used a non-parametric Kruskal–Wallis ANOVA to determine significance. We then used a Dunn’s test with Bonferroni correction to determine the difference between each group.

## Additional files

10.1186/s12977-016-0284-7 Signalling pathways downstream of CCR7. Schematic representation of the signalling pathways activated by PI3K and Ras following chemokine ligation. The site of action and names of specific inhibitors are shown as red lines. Figure is based on [20, 52–54]; and the KEGG Chemokine signalling pathway; http://www.genome.jp/kegg-bin/show_pathway?map04062.
10.1186/s12977-016-0284-7 Dose response of CCL19 on resting CD4^+^ T cells. Resting CD4^+^ T cells were incubated with various concentrations of CCL19 for 5 minutes (A) or 15 minutes (B) and the level of intracellular phosphorylated proteins examined. Cell lysates were assessed by immunobloting using antibody to phosphorylated Akt (pAkt), pNF-κB, pERK, pJNK and loading control GAPDH. Cells treated with PMA and Ionomycin was used as a positive control. Data represent immunoblots of two independent experiments.
10.1186/s12977-016-0284-7 Cytotoxicity of signalling inhibitors on CD4^+^ T cells. Resting CD4^+^ T cells were treated with various inhibitors (see “Methods” for concentrations used) in the presence of CCL19 and incubated for 48 h. Cells were then washed and cultured for another 72 h. Cell viability was determined using live/dead staining and analysed by flow cytometer. Data represents mean ± SD of two independent experiments.
10.1186/s12977-016-0284-7 Integration site selection and gene activation in chemokine treated cells. A, Gene expression was determined by Illumina bead array in unactivated, CCL19-treated or PHA-IL2 activated CD4^+^ T cells after 6 or 72 h. The ratio of expression of genes at the sites of integration was determined in each in vitro condition. B, Expression of individual genes at the site of HIV integration in CCL19-treated resting CD4^+^ T cells (x-axis) compared to unactivated (y-axis; upper panel) or PHA-IL2 activated CD4^+^ T cells (y-axis; lower panel). C, The distance of integration sites to specific genomic elements including LINE, H4K20me3 and H4R3me following HIV infection of unactivated, CCL19-treated and PHA-IL2 activated CD4^+^ T cells, or CD4^+^ T cells from HIV-infected patients on cART or randomly selected sites. Log distance is shown as box plots (median and quartiles) with violin plot of the kernel distribution. The means are shown as a red horizontal line.
10.1186/s12977-016-0284-7Distance from HIV integration sites to genomic and epigenetic marks in CCL19-treated cells (CCL19), other in vitro infected unactivated (Unactive), activated (IL-2/PHA), and in cell from patients on cART (Patient).
10.1186/s12977-016-0284-7. Association between integration sites Alu and LINE in different culture conditions. A, The distance in base pairs from the integration site to the nearest Alu is shown for Alu, Alu S Alu J and Alu Y elements in the human genome. B, Similar analyses for LINE, RTE, CR1, L1 and L2 are shown.
10.1186/s12977-016-0284-7 Common integration sites between cells from different culture conditions. The Venn diagram shows the genes distinct and common between the three culture conditions. The number of genes with insertion sites that were shared between unactivated, CCL19 and PHA-IL2 are indicated. Only 2 genes NDS1 and TBCD (blue) were common with the list of genes with multiple insertion sites reported in CD4^+^ T cells from HIV-infected patients on cART [25–27, 32, 33]. Gene symbols for each of the insertion sites are shown within each group. Genes associated with mitosis, proliferation, or cancer is shown in red.
