# Supplementary figures and images for: HIV integration and the establishment of latency in CCL19-treated resting CD4+ T cells require activation of NF-κB
Source: Retrovirology. 2016 Jul 26;13:49. doi: 10.1186/s12977-016-0284-7 (PMC4962537; doi:10.1186/s12977-016-0284-7)

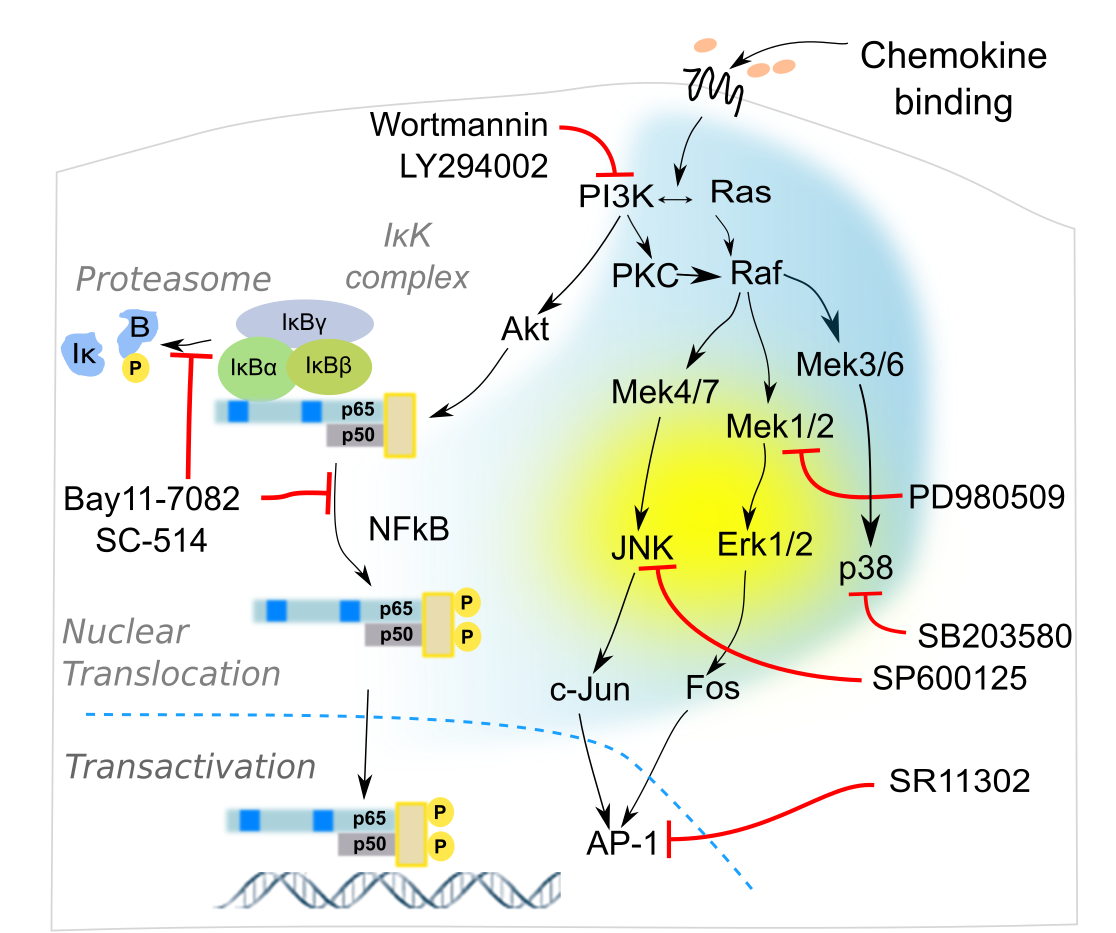

Supplement: Supplementary file 1 — 10.1186/s12977-016-0284-7 Signalling pathways downstream of CCR7. Schematic representation of the signalling pathways activated by PI3K and Ras following chemokine ligation. The site of action and names of specific inhibitors are shown as red lines. Figure is based on [20, 52–54]; and the KEGG Chemokine signalling pathway; http://www.genome.jp/kegg-bin/show_pathway?map04062. [file 12977_2016_284_MOESM1_ESM.tif]

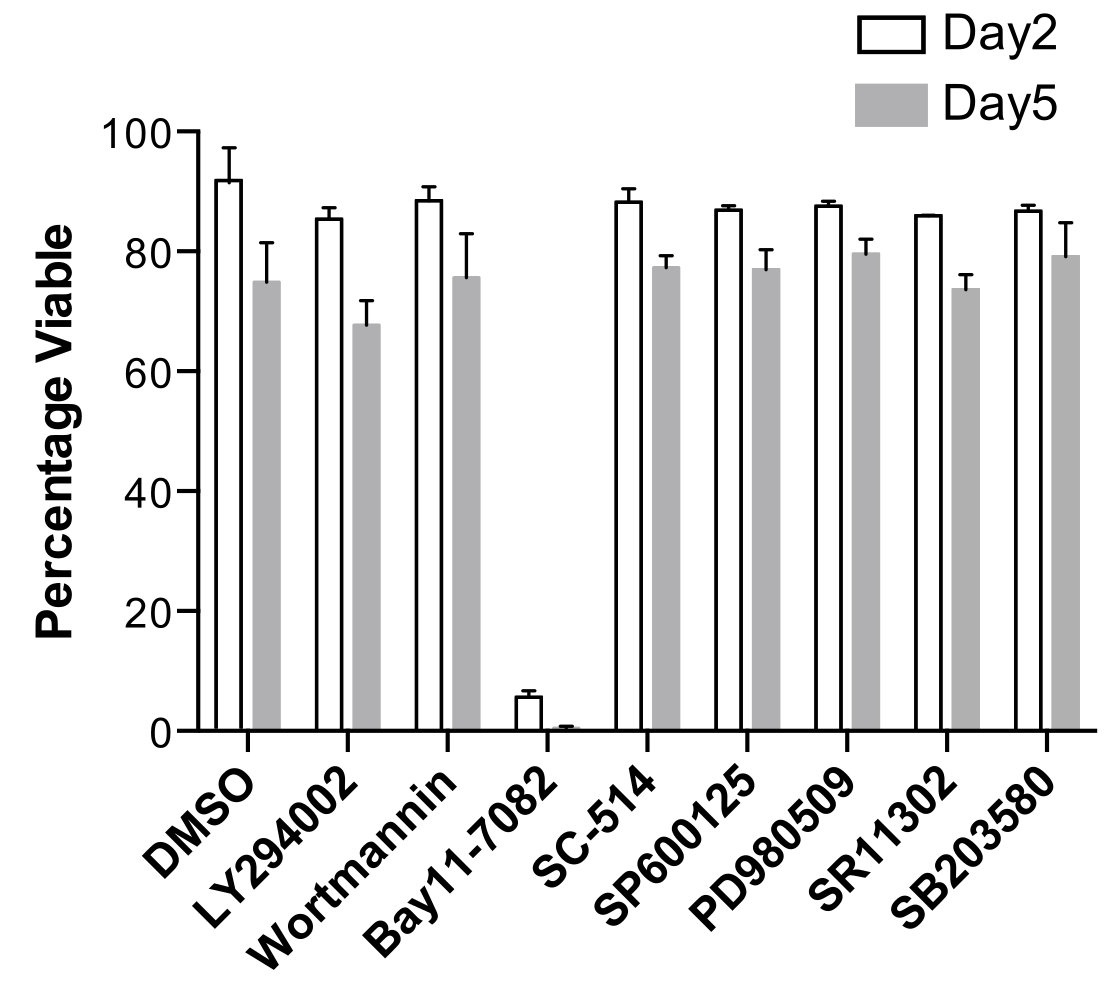

Supplement: Supplementary file 3 — 10.1186/s12977-016-0284-7 Cytotoxicity of signalling inhibitors on CD4+ T cells. Resting CD4+ T cells were treated with various inhibitors (see “Methods” for concentrations used) in the presence of CCL19 and incubated for 48 h. Cells were then washed and cultured for another 72 h. Cell viability was determined using live/dead staining and analysed by flow cytometer. Data represents mean ± SD of two independent experiments. [file 12977_2016_284_MOESM3_ESM.tif]

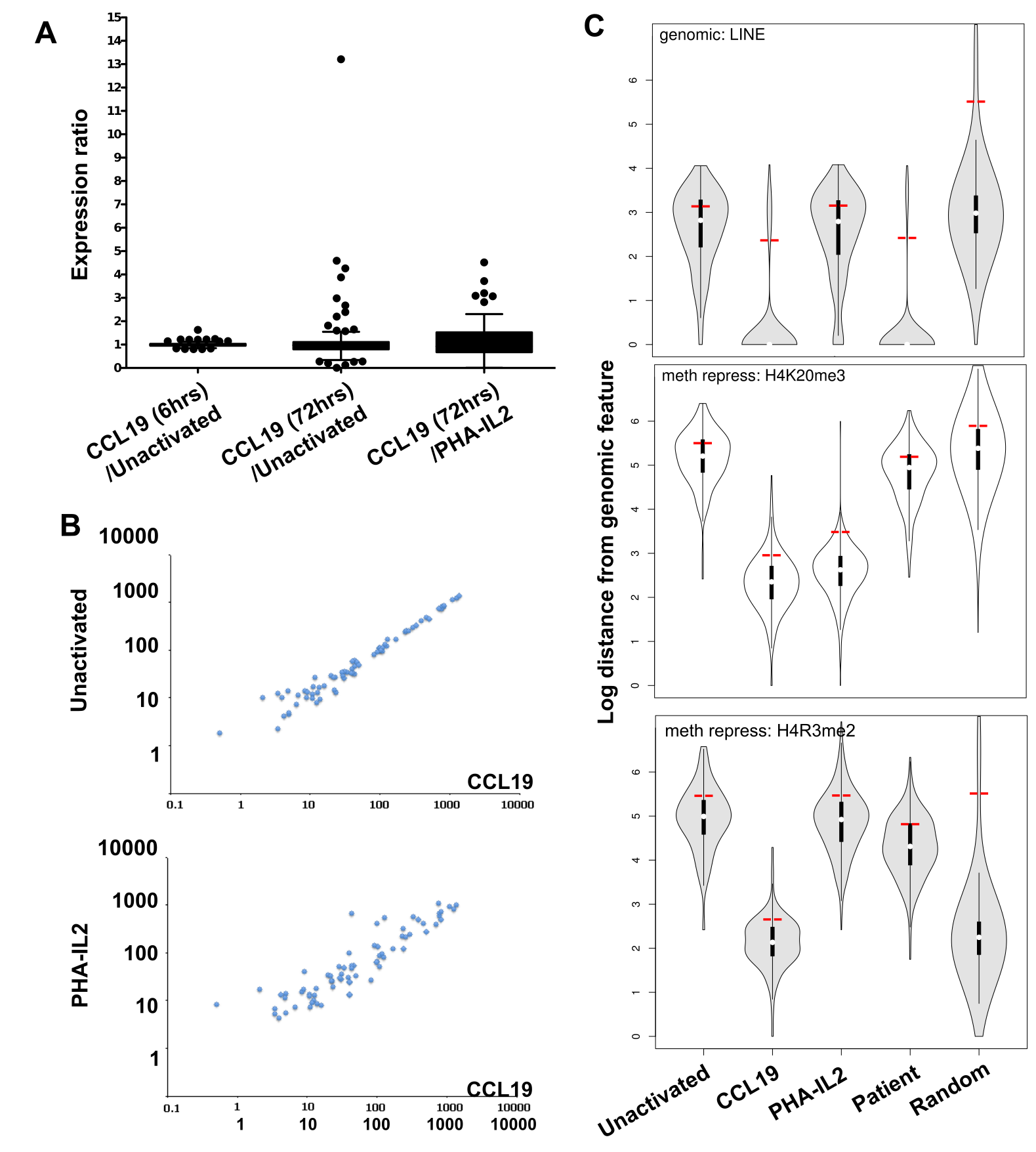

Supplement: Supplementary file 4 — 10.1186/s12977-016-0284-7 Integration site selection and gene activation in chemokine treated cells. A, Gene expression was determined by Illumina bead array in unactivated, CCL19-treated or PHA-IL2 activated CD4+ T cells after 6 or 72 h. The ratio of expression of genes at the sites of integration was determined in each in vitro condition. B, Expression of individual genes at the site of HIV integration in CCL19-treated resting CD4+ T cells (x-axis) compared to unactivated (y-axis; upper panel) or PHA-IL2 activated CD4+ T cells (y-axis; lower panel). C, The distance of integration sites to specific genomic elements including LINE, H4K20me3 and H4R3me following HIV infection of unactivated, CCL19-treated and PHA-IL2 activated CD4+ T cells, or CD4+ T cells from HIV-infected patients on cART or randomly selected sites. Log distance is shown as box plots (median and quartiles) with violin plot of the kernel distribution. The means are shown as a red horizontal line. [file 12977_2016_284_MOESM4_ESM.tif]

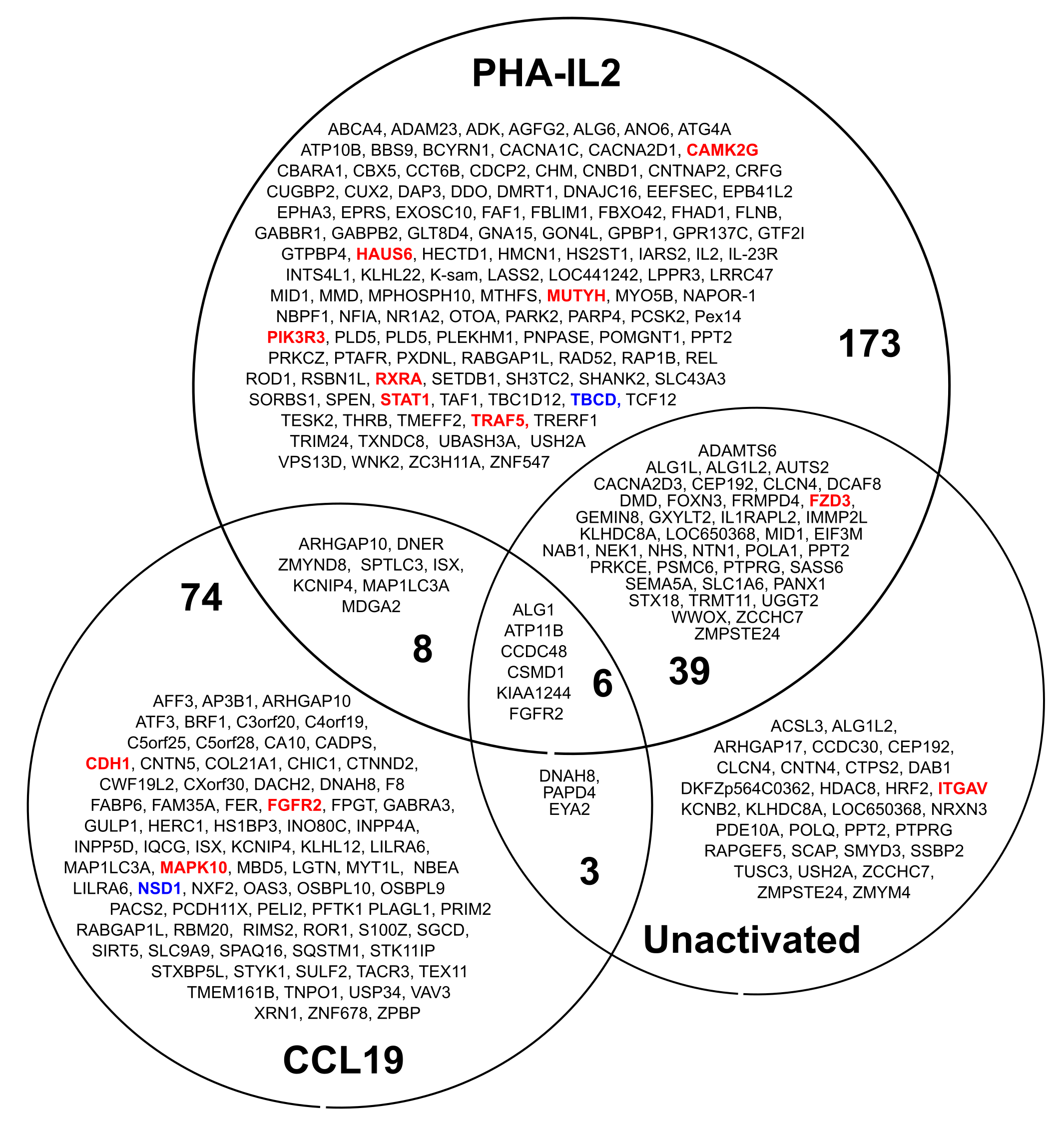

Supplement: Supplementary file 7 — 10.1186/s12977-016-0284-7 Common integration sites between cells from different culture conditions. The Venn diagram shows the genes distinct and common between the three culture conditions. The number of genes with insertion sites that were shared between unactivated, CCL19 and PHA-IL2 are indicated. Only 2 genes NDS1 and TBCD (blue) were common with the list of genes with multiple insertion sites reported in CD4+ T cells from HIV-infected patients on cART [25–27, 32, 33]. Gene symbols for each of the insertion sites are shown within each group. Genes associated with mitosis, proliferation, or cancer is shown in red. [file 12977_2016_284_MOESM7_ESM.tif]
