# Supplementary material for: HIV integration and the establishment of latency in CCL19-treated resting CD4+ T cells require activation of NF-κB
Source: Retrovirology. 2016 Jul 26;13:49. doi: 10.1186/s12977-016-0284-7 (PMC4962537; doi:10.1186/s12977-016-0284-7)
Supplement: Supplementary file 5 — 10.1186/s12977-016-0284-7Distance from HIV integration sites to genomic and epigenetic marks in CCL19-treated cells (CCL19), other in vitro infected unactivated (Unactive), activated (IL-2/PHA), and in cell from patients on cART (Patient). [file 12977_2016_284_MOESM5_ESM.doc]

**Table S1. Distance from HIV integration sites to genomic and epigenetic marks in CCL19-treated cells (CCL19), other *in vitro* infected unactivated (Unactive), activated (IL-2/PHA), and in cell from patients on cART (Patient).**

| **Name** | **Median distance in base pair** | | | | ***Significant (*p* < 0.05)** | | | |
| --- | --- | --- | --- | --- | --- | --- | --- | --- |
|  | **CCL19** | **Unactive** | **IL-2/PHA** | **Patient** | **CCL19 vs Unactive** | | **CCL19 vs IL-2/PHA** | **CCL19 vs Patient** |
| **Genomic features** | | | | | | | | |
| CTCF | 637 | 630 | 575 | 0 | ns | ns | | <0.001 |
| Pol II | 482 | 600 | 471.5 | 53.5 | ns | ns | | <0.001 |
| H2AZ | 137824 | 26286 | 20478.5 | 0 | <0.001 | <0.001 | | <0.001 |
| DNase HS | 371016 | 151024 | 93937.5 | 8974 | <0.001 | <0.001 | | <0.001 |
| CPG | 49952.5 | 52526 | 43503.5 | 0 | ns | ns | | <0.001 |
| TSS | 42464 | 115844 | 35551 | 1260.5 | <0.001 | <0.001 | | <0.001 |
| LINE | 0 | 660 | 620 | 0 | <0.001 | <0.001 | | ns |
| Alu | 237153.5 | 119315 | 109604 | 54660.5 | 0.002 | <0.001 | | <0.001 |
| **Histone methylations associated with transcriptional activation** | | | | | | | | |
| H4K20me1 | 355548 | 106568 | 59156 | 769.5 | <0.001 | <0.001 | | <0.001 |
| H2K8me1 | 236070.5 | 47012 | 30049 | 1150.5 | <0.001 | <0.001 | | <0.001 |
| H3K4me3 | 127236 | 26628 | 21292 | 1353.5 | <0.001 | <0.001 | | <0.001 |
| H3K9me1 | 157819.5 | 35764 | 19077.5 | 687 | <0.001 | <0.001 | | <0.001 |
| H3K27me1 | 170731 | 48269 | 28025.5 | 0 | <0.001 | <0.001 | | <0.001 |
| H3K79me3 | 94983 | 97813 | 32032 | 0 | ns | <0.001 | | <0.001 |
| H3K36me3 | 245433 | 54900 | 33704.5 | 3267.5 | <0.001 | <0.001 | | <0.001 |
| **Histone methylations associated with transcriptional repression** | | | | | | | | |
| H3K27me3 | 226121 | 97813 | 89286 | 55508 | <0.001 | <0.001 | | <0.001 |
| H4K20me3 | 225 | 161132 | 422.5 | 88493.5 | <0.001 | ns | | <0.001 |
| H3K9me2 | 245037.5 | 108528 | 119361.5 | 76752 | <0.001 | <0.001 | | <0.001 |
| H3K9me3 | 655407 | 297680 | 322386.5 | 263599.5 | <0.001 | <0.001 | | <0.001 |
| H3K27me2 | 135601.5 | 67899 | 61737.5 | 52776.5 | 0.02 | <0.001 | | <0.05 |
| H3K27me3 | 226121 | 97813 | 89286 | 55508 | <0.001 | <0.001 | | <0.001 |
| H3K79me1 | 306359.5 | 84121 | 56052.5 | 0 | <0.001 | <0.001 | | <0.001 |
| H3K79me2 | 618866.5 | 232058 | 200058 | 59131 | <0.001 | <0.001 | | <0.001 |
| H4R3me2 | 136 | 98073 | 83415 | 20443 | <0.001 | <0.001 | | ns |
| **Histone methylations associated with intermediate transcriptional activity** | | | | | | | | |
| H3K4me1 | 153195.5 | 26300 | 16816.5 | 868 | <0.001 | <0.001 | | <0.001 |
| H3K4me2 | 174567 | 44822 | 23787 | 847.5 | <0.001 | <0.001 | | <0.001 |
| H3K36me1 | 169178.5 | 43313 | 33773 | 6709.5 | <0.001 | <0.001 | | <0.05 |
| H3R2me1 | 140685.5 | 30132 | 25923 | 5082.5 | <0.001 | <0.001 | | <0.001 |
| H3R2me2 | 313774.5 | 90380 | 76160 | 11702 | <0.001 | <0.001 | | <0.001 |
| **Histone acetylations associated with transcriptional start sites** | | | | | | | | |
| H2BK9ac | 618 | 743 | 662 | 279.5 | ns | ns | | <0.001 |
| H2AK5ac | 649 | 386 | 423.5 | 284 | ns | ns | | <0.001 |
| H3K9ac | 329.5 | 298 | 366 | 105 | ns | ns | | <0.001 |
| H3K14ac | 209 | 277 | 312 | 256 | ns | ns | | ns |
| H3K18ac | 663 | 485 | 442.5 | 97.5 | ns | ns | | <0.001 |
| H3K23ac | 558.5 | 525 | 440 | 276 | ns | ns | | <0.001 |
| H3K27ac | 1174.5 | 736 | 719 | 88.5 | <0.001 | <0.001 | | <0.001 |
| H3K36ac | 334.5 | 270 | 337 | 87 | ns | ns | | <0.001 |
| H4K91ac | 3116.5 | 1051 | 906 | 71 | <0.001 | <0.001 | | <0.001 |
| **Histone acetylations associated with promoter sites** | | | | | | | | |
| H2AK120ac | 962 | 640 | 567 | 440 | ns | <0.05 | | <0.001 |
| H2BK12ac | 785.5 | 432 | 401 | 1521 | <0.05 | <0.05 | | <0.001 |
| H2BK20ac | 729.5 | 618 | 471 | 4379.5 | ns | <0.05 | | <0.001 |
| H3K4ac | 748.5 | 450 | 465 | 81.5 | <0.05 | <0.05 | | <0.001 |
| H4K5ac | 587.5 | 272 | 325 | 116.5 | <0.001 | <0.001 | | <0.001 |
| H4K8ac | 515.5 | 236 | 303 | 747 | <0.001 | <0.001 | | <0.001 |
| H4K12ac | 525 | 394 | 350 | 111.5 | ns | ns | | <0.001 |
| H4K16ac | 138 | 183.5 | 173.5 | 64 | ns | ns | | <0.001 |

*non-parametric ANOVA, Kruskall-Wallis test was used to determine significance. DUNN’S test was used to determine the difference between each group.
